# Fibro-Adipose Vascular Anomaly: A Case Report and Literature Review

**DOI:** 10.7759/cureus.30757

**Published:** 2022-10-27

**Authors:** Bharat Parmar, Jeffrey S Joseph, Kavin Ilangovan G, Alam Khalil-Khan, Rajamani Anand, Ealai A Parthasarathy, Moien AB Khan

**Affiliations:** 1 Department of Radiology, Chettinad Hospital and Research Institute CARE, Kelambakkam, Chennai, IND; 2 Medicine and Surgery, Government Medical College, Omandurar, Chennai, IND; 3 Department of Academic Unit of Primary Medical Care, The University of Sheffield, Sheffield, GBR; 4 Department of Family Medicine, College of Medicine and Health Sciences, United Arab Emirates University, Al Ain, ARE; 5 Department of Primary Care, North West London - National Health Service Provider, London, GBR

**Keywords:** mobility limitation, unilateral leg swelling, venous malformation, vascular malformation, dilation of veins

## Abstract

A fibro-adipose vascular anomaly (FAVA) is a complex venous malformation characterized by intramuscular fibrofatty replacement and dilation of veins. As FAVA is a rare entity and associated with a complex constellation of vascular anomalies, it is often misdiagnosed. This report discusses a case of a 26-year-old woman who presented with swelling on the lateral aspect of the right thigh. FAVA was diagnosed on the basis of radiological and histopathological examinations. After en-bloc resection of the mass, the patient's pain and ability to move significantly improved. We describe the clinical, radiological, and pathological aspects of FAVA, as well as its management.

## Introduction

A fibro-adipose vascular anomaly (FAVA) is a complex vascular anomalous malformation that has been recently described. It most commonly affects young women, with a female-to-male ratio of 3:1; however, it can affect anyone from 1 to 30 years of age [1]. FAVA is characterized by phlebectasia and fibrofatty tissue replacement of muscle, as well as lymphatic involvement and venous thrombosis. Alomari et al. first described the lesions in 2014 [2,3], but the International Society for the Study of Vascular Anomalies (ISSVA) revised their classification in 2018 and categorized them as distinct entities. Most of the lesions are sporadic and caused by somatic mutations in the phosphatidylinositol-4,5-bisphosphate 3-kinase catalytic subunit alpha (PIK3CA) gene [2]. The PIKC3A genes are associated with lymphatic and venous malformations [4]. Due to the rarity of the condition, establishing an accurate diagnosis is challenging when compared with other vascular anomalies.

## Case presentation

A 26-year-old female presented to the hospital with swelling over the lateral aspect of the right thigh for more than 10 years. It had an insidious onset and had progressed gradually, accompanied by severe local pain and restricted mobility. For the last three months, she had been experiencing increasing pain and discomfort in her right thigh. Moreover, the patient had been involved in a road traffic accident three years ago, sustaining injuries to the right thigh, pelvis, and right hand. Plating, open reduction, and internal fixation with interlock nails had been performed to treat the patient's shaft fracture of the femur and transverse fracture of the acetabulum. There were no abnormal findings in hematological investigations, including coagulation profile and inflammatory markers. The right lower limb appeared leaner on inspection. Upon palpation, the right thigh had a lumpy, spongy swelling. The range of motion was limited, with flexion limited to 40 degrees, and the pain further restricted it. The lower leg was examined and showed normal motor power and skin. Approximately 5 cm of healed surgical scarring was found on the proximal aspect of the right thigh.

**Figure 1:**
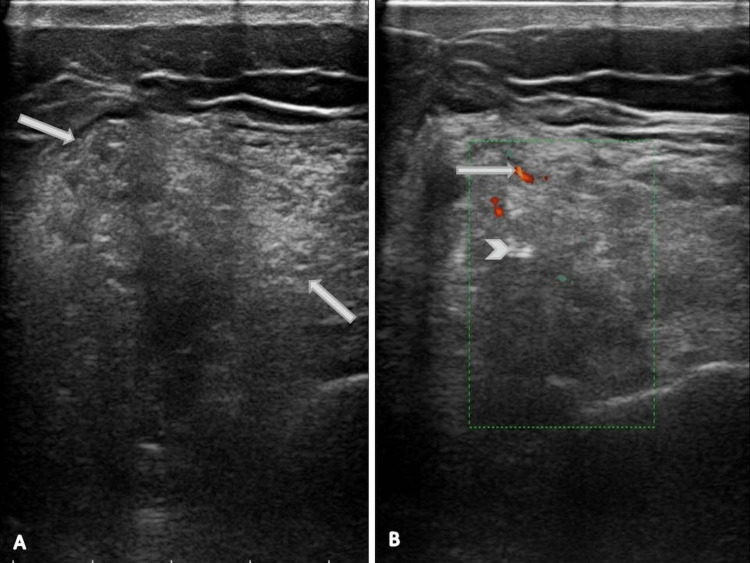
Ultrasound shows (A) a well-defined, spindle-shaped, predominantly echogenic lesion without compressible venous channels. (B) Colour doppler shows minimal venous flow. The lesion shows the presence of calcification

Phleboliths were not visible on an X-ray. An ultrasound was performed for swelling in the right lateral aspect of the thigh, and fatty changes were observed in the intramuscular plane of the lateral aspect of the right mid and distal thigh (Figure 1A). There were a few anechoic serpiginous channels on color Doppler with low venous waveform velocity. The study also revealed echogenic foci with posterior acoustic shadows suggesting calcifications (Figure 1B).

**Figure 2:**
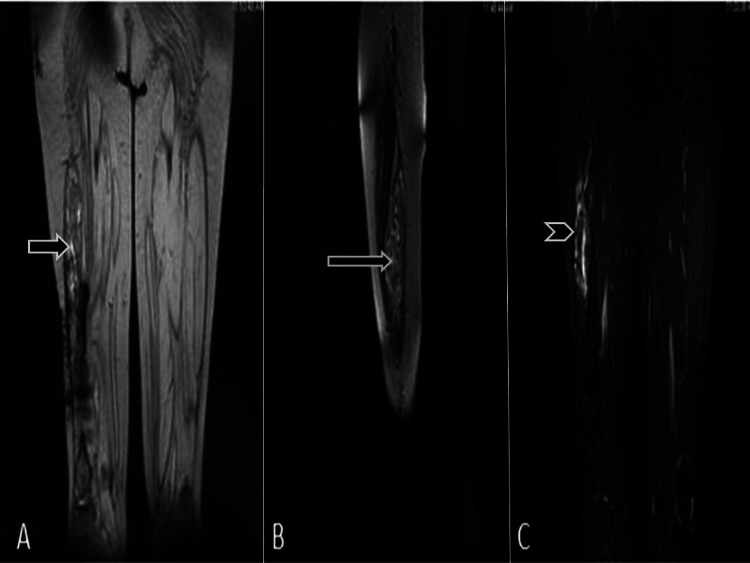
MRI of the patient Images showing coronal T2 (A) T2 sagittal (B) and STIR sequences (C) that predominantly show hyperintense lesions with a fatty component in the right thigh, which is suppressed on STIR. Proximal femur fracture with artifacts from plate and screw fixation is also seen. Diffuse atrophy of all muscles of the proximal thigh is noted MRI: magnetic resonance imaging; STIR: short-tau inversion recovery

An MRI scan of the right thigh revealed heterointense, predominantly hyperintense, lobulated lesions within the vastus lateralis muscle (Figures 2A, 2B). Short tau inversion recovery (STIR) with fatty components showed suppression (Figure 2C).

**Figure 3:**
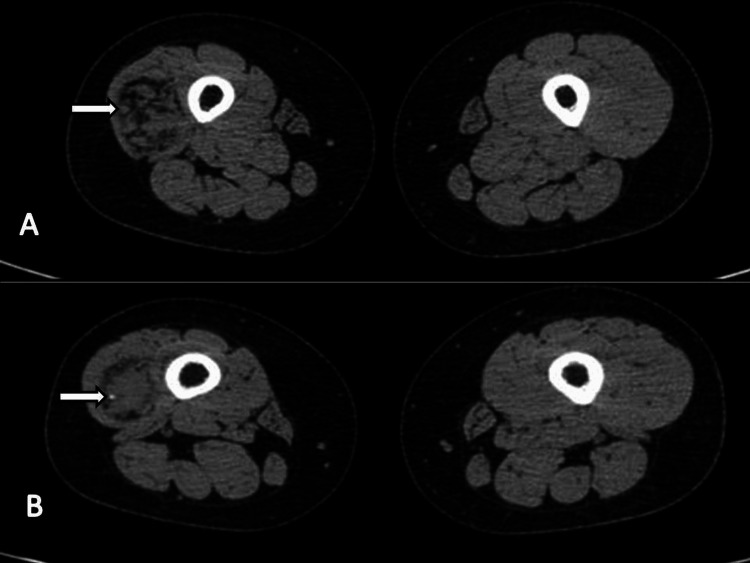
Non-contrast CT images The images show circumscribed heterodense lesion with fat density in intervening spaces on the axial plane (A) and a few calcified phleboliths in the inferior aspect of the lesion (B) CT: computed tomography

There were multiple hypointense T1/T2 areas in the lesion. MRI revealed atrophy and fatty replacement of vastus lateralis muscles. CT without contrast showed circumscribed heterodense intramuscular lesions with fat density in the intervening spaces (Figure 3A). The inferior aspect of the lesion showed a few calcified foci, suggesting phleboliths (Figure 3B).

**Figure 4:**
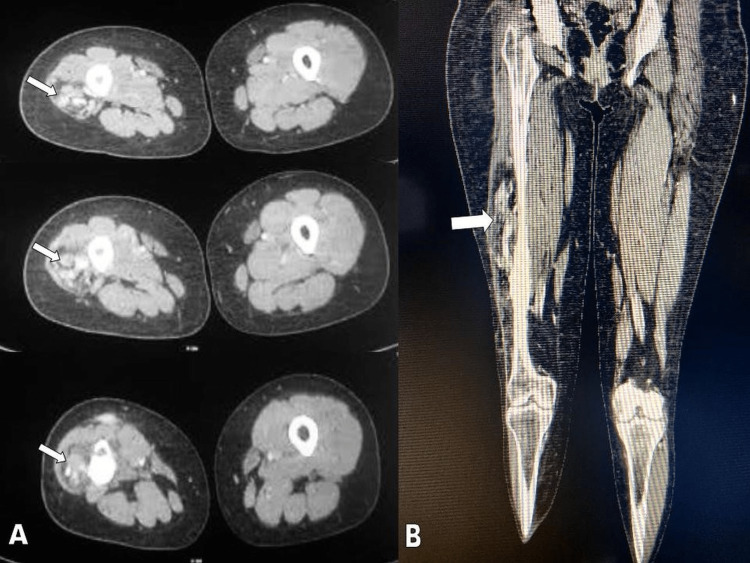
CT venogram A circumscribed heterodense intramuscular mass within vastus lateralis muscle measuring 2.0 (anteroposterior) x 4.5 (craniocaudal) x 3.5 (transverse) cm, with multiple enhancing tortuous vessels (white arrows) is seen in the axial plane of the right thigh (A). Coronal plane (B) showed diffuse atrophy of muscles of the proximal thigh with regional fatty replacement of vastus lateralis muscle (white arrow) CT: computed tomography

**Figure 5:**
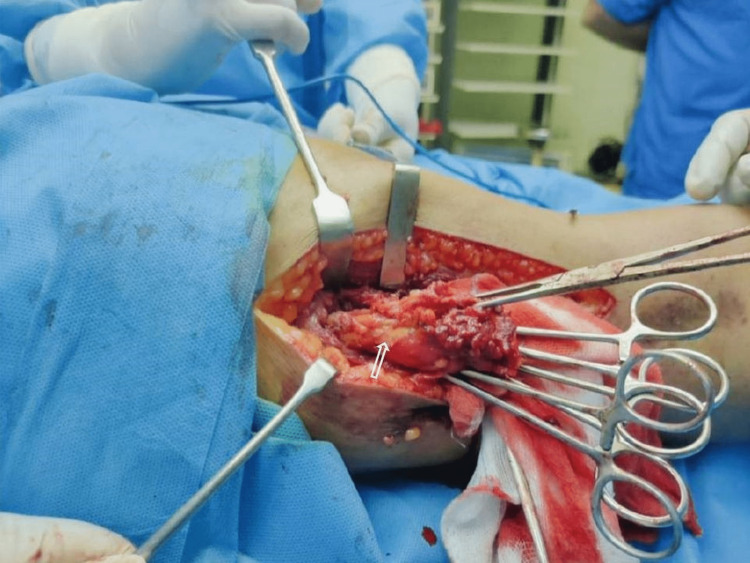
Intraoperative image after the exploration of the lateral aspect of the thigh demonstrating typical macroscopic fibrofatty change of the involved musculature (white arrow)

A CT venogram (Figures 4A, 4B) showed multiple enhancing tortuous veins within the vastus lateralis muscle. During the delayed phase, contrast accumulated in the lesion. There was no washout during the delayed phase. It was observed that some ectatic vessels were communicating with the superficial femoral vein. The vastus lateralis muscle was replaced with fat. As a result of the lesion, there was no increased arterial flow or arteriovenous shunting. A differential diagnosis was considered for FAVA based on the clinical and radiological features. The patient underwent an en-bloc excision of the entire lesion (Figure 5).

**Figure 6:**
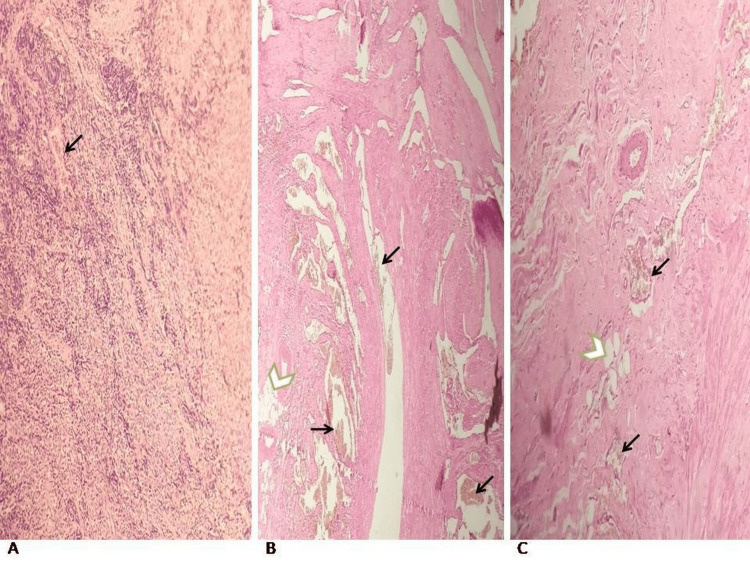
Histopathology images with H & E stain A. The image shows revealed fibromuscular tissue with congested blood vessels showing thrombi (black arrow). B and C. The images show fibro-adipose tissue (green arrowheads) with numerous closely packed thick-walled blood vessels of varying sizes; most of them were dilated and showed congestion (black arrows). Focal lymphoplasmacytic infiltrates were also noted

Figure 5 illustrates the intraoperative findings of a fatty mass entwined with muscle tissue and several large veins in the right vastus lateralis muscle. Histopathology was performed on the specimen. A few large veins were interspersed with fat and fibrous tissue in the specimen. A histological section revealed fibromuscular tissue with congested blood vessels showing microthrombi (Figure 6A). Fibro-adipose tissue with a large number of thick-walled blood vessels of different sizes, most of which were dilated and congested, was seen. Furthermore, lymphoplasmacytic infiltrates were also present (Figures 6B, 6C).

A positive clinical outcome was achieved, and the patient was discharged with recommendations for physical therapy and pain medications. At the six-month and one-year follow-ups, the patient showed remarkable improvements without pain at the site of surgery and had regained full range of motion.

## Discussion

In 2014, Alomari et al. described FAVA as a constellation of vascular anomalies with distinct clinical, radiological, and histopathological characteristics [3]. FAVA is characterized by fibrofatty infiltration of muscles, dilated veins, contractures of affected extremities, and persisting pain. A recent classification by the ISSVA categorizes it as a separate entity under provisionally unclassified vascular anomalies. The unclassified categories include sinusoidal haemangioma, angiokeratoma, phosphatase and tensin homolog (PTEN) hamartoma of soft tissues (PHOST), acral arteriovenous tumor, and intramuscular hemangioma. In addition to reticulate capillary malformations of megalencephaly-capillary malformation-polymicrogyria (MCAP), PIK3CA gene-associated vascular anomalies include cystic lymphatic and vascular malformations, Klippel-Trenaunay syndrome, congenital lipomatous overgrowth, epidermal nevi, and CLAPO syndrome (Capillary vascular malformation of the lower lip, Lymphatic malformations of the head and neck, Asymmetry and Partial or generalized Overgrowth).

Several closely associated vascular anomalies should be distinguished from each other first. A recent case series indicated a female predilection and an age range of 1-30 years, with the thigh being the second most common site after the calf [3,5,6]. There is a distinction between FAVA and congenital vascular malformations (CVM), which are classified under separate vascular anomalies. The prevalence of CVM is equal among men and women, while the prevalence of FAVA is more common among women. In FAVA, atrophied skeletal muscles are characterized by abnormal fibrous and fatty tissue, along with abnormal ectatic venous channels, occasional thrombi, and lymphoplasmacytic aggregates.

FAVA and CVM differ in several ways. In FAVA, the quadriceps and intrinsic foot muscles are involved, while in CVM, the gastrocnemius and wrist muscles are involved. Thighs are the second most common site, followed by calves [3,6]. FAVA can be classified as an infiltrative lesion, a mass-like lesion, or a diffuse infiltrative lesion. When present, CVM is usually asymptomatic but can cause occasional episodic pain. On ultrasound, FAVA appears as a hyperechoic lesion with dilated veins, while CVM appears as blood-filled, compressible spaces. FAVA is characterized by dense fatty infiltrates, whereas CVM, on the other hand, is characterized by mainly spongiform features. The MRI of FAVA shows heterogeneous, diffuse moderate-to-strong enhancement, whereas CVM appears as a fluid signal and phleboliths as a patchy heterogeneous enhancement. A PHOST must also be differentiated. It is common for patients with PHOST to also have symptoms of a syndromic PTEN-related disease, such as Bannayan-Riley-Ruvalcaba syndrome or Cowden syndrome. In contrast with FAVA, which does not exhibit any syndromic characteristics, PHOST can be syndrome-related and characterized by fibrous and adipocytic tissues, thick-walled arteries, multiple small vessels, venous channel clusters, lymphoid follicles, and occasionally arteriovenous communication. It is common for FAVA lesions to affect only a single intramuscular compartment without crossing the fascial planes, while PHOST lesions usually exhibit a trans-spatial involvement with inconspicuous margins. There is also a vivid enhancement of PHOST due to the presence of mass. PHOST lesions have a smaller venous component on ultrasound and a relatively smaller hypervascular component on spectral imaging. A closely related pathological entity is an intramuscular hemangioma, which causes contractures similar to FAVA [7,8].

In addition to observation and conservative treatment, FAVA can be managed with physical therapy, splints, casts, sclerotherapy, intralesional steroid injections, cryoablations, and, more recently, sirolimus. The surgical excision of this condition is a good long-term curative option, despite its difficulty with larger, more infiltrative, or deeper lesions. Moreover, it provides excellent results in terms of reducing pain and regaining movement limitations. There are several options available for patients with extensive muscle involvement and nerve entrapment, including compartment decompression, partial resection, neurolysis, tendon reconstruction, and free functional tendon transfer [9,10,11]. Sclerotherapy may reduce pain in lesions with larger veins; however, it may not be effective in the long term in masses with more solid tissue. In children, sirolimus has been shown to be effective in treating complicated vascular anomalies [9,10]. Despite its success in treating complicated vascular anomalies, large studies have not been conducted to document its effectiveness. As a result of its immunosuppressive properties, its use should only be considered in consultation with a physician who is an expert in this field. Cryoablation has been used to control pain as a treatment option for FAVA. Although it is a minimally invasive procedure, it is not widely available. Moreover, the long-term outcomes and complications of cryoablation are not well documented, and hence further research is needed. In addition, it is only effective for superficial, well-circumscribed lesions, and not for deep, infiltrating lesions.

Perineural scarring can result from fibrofatty infiltration, causing contractions and motor dysfunction, neurogenic pain, and hyperesthesia. In our case, the intramuscular lesions caused functional limitations, while the anomalous veins contributed to swelling, deformity, and focal pain. The deformity may be due to pain and contracture. Focal pain may be the result of neural stimulation or infiltration by fibro-adipose venous components.

## Conclusions

FAVA is a rare and newly described entity with distinctive clinical, radiological, and pathological characteristics. It is imperative that clinicians have a thorough understanding of FAVA in order to provide proper diagnosis and treatment referrals. FAVA is often misdiagnosed with other congenital vascular malformations. FAVA is a rare, but specific vascular malformation that poses significant management challenges. Multidisciplinary teamwork is essential in its management. In spite of the loss of muscle bulk and function associated with surgery, pain is reduced and deformities are corrected, which improves function.
